# Drug repositioning as a route to anti-malarial drug discovery: preliminary investigation of the *in vitro* anti-malarial efficacy of emetine dihydrochloride hydrate

**DOI:** 10.1186/1475-2875-12-359

**Published:** 2013-10-09

**Authors:** Holly Matthews, Maryam Usman-Idris, Farid Khan, Martin Read, Niroshini Nirmalan

**Affiliations:** 1 School of Environment and Life Sciences, University of Salford, M5 4WT, Salford, Manchester, UK; 2 Lumophore Ltd. Manchester Science Park, M15 6SE, Manchester, UK; 3 Manchester Institute of Biotechnology, University of Manchester, M1 7DN, Manchester, UK

**Keywords:** Drug repositioning, Antimalarial chemotherapy, Emetine, Dihydroartemisinin, Drug susceptibility assays, Flow cytometry

## Abstract

**Background:**

Drug repurposing or repositioning refers to the usage of existing drugs in diseases other than those it was originally used for. For diseases like malaria, where there is an urgent need for active drug candidates, the strategy offers a route to significantly shorten the traditional drug development pipelines. Preliminary high-throughput screens on patent expired drug libraries have recently been carried out for *Plasmodium falciparum*. This study reports the systematic and objective further interrogation of selected compounds reported in these studies, to enable their repositioning as novel stand-alone anti-malarials or as combinatorial partners.

**Methods:**

SYBR Green flow cytometry and micro-titre plate assays optimized in the laboratory were used to monitor drug susceptibility of *in vitro* cultures of *P. falciparum* K1 parasite strains. Previously described fixed-ratio methods were adopted to investigate drug interactions.

**Results:**

Emetine dihydrochloride hydrate, an anti-protozoal drug previously used for intestinal and tissue amoebiasis was shown to have potent inhibitory properties (IC_50_ doses of ~ 47nM) in the multidrug resistant K1 strain of *P. falciparum*. The sum 50% fractional inhibitory concentration (∑FIC_50, 90_) of the interaction of emetine dihydrochloride hydrate and dihydroartemisinin against the K1 strains of *P. falciparum* ranged from 0.88-1.48.

**Conclusion:**

The results warrant further investigation of emetine dihydrochloride hydrate as a potential stand-alone anti-malarial option. The interaction between the drug and the current front line dihydroartemisinin ranged from additive to mildly antagonistic in the fixed drug ratios tested.

## Background

The widespread emergence of anti-malarial drug resistance has rendered monotherapy regimes largely ineffective [1]. The World Health Organization recommendation for combination therapy with the use of artemisinins is aimed at impeding the development of resistance to the current frontline drug [2]. However, early resistance to artemisinins has been reported in the field and generated readily in the laboratory setting [3,4]. Furthermore, resistance to the majority of potential partner drugs available for artemisinin combination therapy (ACT) significantly limits combinatory options [5,6]. The urgent need to develop novel, potent anti-malarials as well as synergistic partners for artemisinins and ACT cannot be overemphasized.

Reliance on the traditional drug development pathways to deliver on this goal would have significant implications on both cost and time. Drug repositioning or the screening of existing drugs for new uses, affords an attractive, alternate and valid paradigm for drug discovery [7,8]. Recent successes like the repositioning of Viagra® for erectile dysfunction and Thalidomide® for Erythema nodosum leprosum, have lead drug companies to explore repositioning on a more systematic basis [9,10]. Given that 90% of drug candidates fail during development, this approach which utilizes bioactive compounds with known safety profiles must necessarily be advantageous [11,12]. For diseases like malaria, drug repositioning may not only deliver novel candidates, but also provide partner drugs for combinatorial regimes with artemisinins, thereby increasing longevity of this highly effective and affordable frontline drug [13,14]. The void in the market for new anti-malarial drug classes and the lack of affordable alternatives in the developmental pipeline, make it imperative that faster drug developmental processes are urgently sought to avoid the imminent, potentially catastrophic consequences of drug failure. Patent expired drug compound libraries, such as the Library of Pharmaceutically Active Compounds (LOPAC: Sigma), have already been screened for anti-malarial activities and potential candidates identified [15]. This work and other screening initiatives have yielded a large compliment of anti-malarial drug candidates which are now available in the public domain in order to enable a more rigorous definition and characterization of their anti-malarial efficacies. Against a backdrop of emerging artemisinin resistance and a fast-depleting armamentarium of affordable anti-malarial therapeutic options, it is critical that candidates from such preliminary screening initiatives are further investigated objectively and systematically to evaluate their therapeutic potential.

The work presented here follows on from data published from a high-throughput anti-malarial screening initiative on three compound libraries, namely the Library of Pharmaceutically Active Compounds (LOPAC; Sigma), the library from the National Institute of Neurological Disorders and Stroke (NINDS) and the Library of uncharacterized compounds (Chembridge) [15]. The high-throughput 384-well luciferase screen on 12,320 compounds at 5.5 μM concentrations yielded a total of 163 compounds exhibiting an 85% reduction in parasitaemia in the drug-sensitive 3D7 strain of *P. falciparum*.

The objective of this study was the selective corroboration of some of the candidates identified in the Lucumi study and the further definition/characterization of these leads to identify stand-alone anti-malarial options and potential synergistic candidates for artemisinins [15]. This second phase screening was carried out on the multidrug resistant K1 strains of *P. falciparum* using a more robust drug susceptibility assay. SYBR green fluorescence-based micro titre plate and flow cytometric assays were optimized to map drug susceptibility. This versatile DNA-based screening technique is ideally suited for *P. falciparum* due to its location within an enucleate red blood cell and provides an objective and reliable method to study pharmacodynamics in an in depth manner. Emetine dihydrochloride hydrate was selected for further investigation of its anti-malarial properties based on the inferences from the preliminary screens of the LOPAC library.

The significant advantages of combination therapy have been clearly demonstrated in recent clinical trials conducted in areas of drug-resistant malaria in Africa [16-18]. The preliminary work reported here provides a more in depth pharmacodynamic perspective of the anti-malarial efficacy of emetine as a stand-alone anti-malarial and a combinatorial partner with dihydroartemisinin. The work justifies the further analysis of the anti-protozoan drug as a valid option for repurposing/repositioning in malaria.

## Methods

### Parasite culture

*Plasmodium falciparum* parasites (strain K1) were maintained routinely in complete RPMI 1640 medium containing L-glutamine (+) 25 mM Hepes (Gibco, Life Technologies, UK) supplemented with 5 mg/L albumin bovine serum fraction V (Sigma, UK), 50 mg/L hypoxanthine (Sigma, UK), 5 ml/L of 40% glucose (Dextrose Anhydrous, Fisher Scientific, UK) and 50 mg/L of gentamycin (Sigma, UK) in PBS. The parasites were constantly maintained in O + blood in accordance with the methods of Read and Hyde (1993) [19]. Whole blood was centrifuged at 3,000 rpm (4000 g) for 5 minutes at room temperature and the buffy coat removed. The process was repeated twice after re-suspension in 1640 RPMI to ensure complete removal of white blood cells. Washed blood was stored at 4°C as a 50% haematocrit in complete RPMI medium. Parasites were cultured continuously in 25 or 12.5 cm^2^ flasks in final culture volumes of 10 ml and 5 ml respectively and maintained at 5% final haematocrit. Subcultures where completed at either 48 or 72 hour intervals. Sorbitol synchronization was carried out prior to experiments, as described previously [19,20]. Briefly, sorbitol solution (5% w/v in distilled water and filtered through a 0.22 μm filter) was added to the parasite pellet and incubated for 5 mins. The culture was centrifuged at 3,000 rpm for 5 minutes and the supernatant discarded. The pellet was washed three times in complete RPMI prior to re-suspension at the appropriate haematocrit. Giemsa-stained thin blood smears were made to determine parasitaemia before sub-culture and prior to experimental set-ups. Cultures were initiated at a starting parasitaemia of 0.5%. Flasks were gassed with a 5% CO_2,_ 5% O_2,_ 90% N_2_ air mixture (BOC Limited, UK) and incubated in the dark at 37°C (Leec culture safe touch 190 CO_2,_ Leec Limited, UK).

### Giemsa microscopic test

A thin smear was prepared, air dried at room temperature and fixed in 100% methanol. The slide was stained for 20 min in Giemsa stain (BDH/WVR UK) diluted 1:10 in Giemsa buffer (BDH, UK). Parasitaemia was estimated by counting the percentage infected cells per field of view. For each slide, at least three fields of view were counted from which the average percentage of infected cells was calculated.

### Optimization of the SYBR green micro titre plate assay

In order to optimize the SYBR Green micro-titre plate assay, fluorescence intensity reading was correlated with parasite density. In brief, spent media was removed from a continuous culture and the parasitaemia was determined by blood smear. The parasitized blood (50% haematocrit) was diluted with RPMI 1640 to either 10% or 5% haematocrit before transfer in duplicate (200 μl per well) to a 96 well plate. A non-infected blood sample (5% haematocrit) was also added in duplicate and served as a negative control. Two fold serial dilutions were then performed using 100 μl of RPMI 1640 leaving a final volume of 100 μl per well. Additional controls included wells containing 100 μl of either RPMI 1640 or complete media (6 wells per media solution). Finally, 100 μl of 2.5 x SYBR Green in RPMI 1640 was added to each well and the plate was incubated for 1 hour at room temperature (diluting starting haematocrits to 5% and 2.5% repectively). Fluorescence intensity was measured from above using a GENios plate reader (Tecan) with excitation and emmision wavelenghts of 485 nm and 535 nm respectively. Default settings of the Magellan software programme for em485/ex535 fluorescence were employed. Gain settings of the instrument were adjusted to a value of 80. Absolute fluorescence values for each well were recorded. There were dulplicate wells for each dilution and the experiment was repeated twice.

### Optimized SYBR green micro-titre plate assay for *P. falciparum*

Following drug treatment, 5 ml of parasite culture was centrifuged at 14,000 g for 90 seconds and complete media replaced with an equivalent volume of RPMI 1640 to maintain a 5% haematocrit. A sample (100 μl) from each treatment flask was transferred to a 96 well plate (Nunc, Denmark) in triplicate. Controls included non-drug treated, infected and uninfected blood. SYBR Green1 nucleic acid gel stain (10,000 x, Sigma, UK) was diluted 2.5 x working solution in PBS and 100 μl added to each well, giving a total well volume of 200 μl and a final haematocrit of 2.5%. Following a one-hour incubation period at room temperature the plate was viewed immediately as described previously. The average absolute fluorescence values of triplicate wells for each condition were used to generate dose response curves. Three independent experiments were completed.

### SYBR green flow cytometry assay for *P. falciparum*

Following the drug treatment procedure, 50 μl of a 5% haematocrit culture was transferred into fresh Eppendorf tubes. After a single washing step in PBS (centrifugation at 14,000 rpm for 90 secs) each pellet was re-suspended in I ml of 2.5 x SYBR Green1 solution and incubated in the dark for 20 mins at room temperature. Subsequently, the samples were centrifuged (14,000 rpm, 90 secs) and re-suspended in 250 μl of 0.37% formaldehyde solution in PBS (36% molecular biology grade, Sigma, UK). Following fixation, the samples were washed 3 x in PBS and re-suspended in 1 ml of PBS. Fifty thousand events were recorded for each sample using the FITC channel (Blue laser, excitation laser line 488 nm EX_max_ 494 nm/Em_max_520nM) of the BD FACSVerse flow cytometer system. Scatter plots were automatically generated by the BDFACSuite software. FITC fluorescence was plotted against forward scatter and gating was conducted using standardized procedure. Percentage data was then obtained for fluorescent events (within the gates) relative to the total number of events recorded, and used to plot dose response curves.

### Preliminary drug screening for anti-malarial activity

Five compounds, previously reported by Lucumi *et al.*[15] to be potent against *P. falciparum* strain 3D7 (IC_50_ ≤ 5 nM) were taken forward for preliminary screening against the drug resistant K1 strains. The compounds: Emetine dihydrochloride hydrate, SKF 95282 dimaleate, S (−)-UH-301 hydrochloride, Vinblastine® and Vincristine® were selected from the Library of pharmaceutically active compounds (LOPAC: Sigma Aldrich). The LOPAC libraries were stored at −20°C in a 96 well plate format at a concentration of 1 mM. Working stocks were prepared by diluting 1:10 with DMSO, and test concentrations prepared by further dilution with RPMI 1640. Infected blood was diluted to ~ 0.5% parasitaemia and subdivided into 5 ml treatment flasks at 5% haematocrit. Parasites were then treated with the respective IC_50_ of each compound [15] and 10x the IC_50_ to account for the resistance phenotype of the K1 strain. LOPAC compounds were either administered alone or in combination with dihydroartemisinin (DHA). For the preliminary combination assays LOPAC compounds at IC_50_ were used with DHA 0.63 nM or 1.25 nM. The LOPAC 10x IC_50_ treatments were co-administered with 0.63 nM DHA only to enable the combinatory effects to be monitored. Treated and control flasks were incubated under conditions described previously for 48 hours and analysed using the SYBR Green flow cytometer method.

### Drug preparation

Dihydroartemisinin and emetine dihydrochloride hydrate were obtained from Sigma Aldrich. Stock solutions were prepared in DMSO at 5 mM, aliquoted and stored at −20°C. During parasite treatment the stock solution was serially diluted using RPMI to 5 μM. Appropriate volumes of the diluted stock solution were subsequently inoculated into 5 ml of parasite culture flasks to obtain the required test concentrations. Final test concentrations were within the 1 nM – 40 nM and 1 nM - 1000 nM range for DHA and emetine dihydrochloride hydrate respectively (Note: DMSO concentrations did not exceed 0.002%).

### *In vitro* drug interaction assay

To investigate whether the combined effects of emetine hydrochloride hydrate were synergistic, additive or antagonistic, a previously described fixed ratio assay was employed [21]. Dihydroartemisinin and emetine dihydrochloride hydrate were combined in four fixed ratios 4:1, 3:2, 2:3 and 1:4. In addition, each drug was administered alone for direct comparison with the combinations, hereafter referred to as the 5:0 and 0:5 ratios. Approximately eight-fold IC_50_ values were used as 100% (8 x IC_50_ DHA = 20 nM and 8 x IC_50_ Eme = 400 nM). Thus for the first dilution the combinations were as follows for DHA (nM) : Eme (nM) 20:0, 16:80, 12:160, 8:240, 4:320 and 0:400 respectively. For each dilution thereafter drug concentrations were serially diluted two-fold. The IC_50_ for each compound (at the 5:0 and 0:5 ratios) therefore lay within the fourth dilution. Once drug stocks had been prepared in RPMI 1640, trophozoite stage parasites were diluted to ~0.5% parasitaemia and transferred into individual 5 ml treatment and control flasks at 5% haematocrit. Parasites were then treated with the various drug combinations, gassed (as described previously) and incubated for 48 hours at 37°C. Duplicate preparations were set up for each ratio at each dilution. Following treatment, samples were then analysed using the SYBR Green flow cytometry method. Giemsa staining of thin blood smears was also employed to permit parasite stage confirmation.

### *In vitro* stage specific effects of dihydroartemisinin and emetine dihydrochloride hydrate

Parasites were treated with either IC_50_ DHA, IC_50_ emetine, or a combination of both compounds (IC_50_ DHA + IC_50_ Eme). Duplicate treatments were initiated at late trophozoite stage and carried out as described previously. Stage specific effects were analysed for untreated control cultures in parallel to drug treatments at 24, 48 and 72 hour time points. In brief, SYBR Green flow cytometry was used to differentiate between mononuclear and multinuclear parasite forms. The proportion of multinuclear cells was then displayed as a percentage of the total number of parasitized cells recorded for each treatment at each time point.

### Calculation of IC_50_ and IC_90_ values

Data from the Giemsa, SYBR Green micro titre plate and SYBR Green flow cytometry assays were compared. For all data sets the infected blood controls were set at 100% and percentage parasitaemia for drug treated samples was calculated relative to the infected control. For IC_50_ and IC_90_ calculations data was further processed using Graphpad prism 5.0. Data was normalized so that the largest value in the data set corresponded to 100% and the smallest value corresponded 0%. Log-transformed drug concentrations were then plotted against the dose response and the IC_50_ and IC_90_ values were determined using non-linear regression (Graphpad prism 5.0).

## Results

Lucumi *et al.* reported the high-throughput anti-malarial screening of 12,320 compounds from the LOPAC, NINDS and Chembridge libraries using a luciferase assay on the 3D7 strain of *P. falciparum*[15]. Preliminary screens were carried out on drug resistant K1 strains of *P. falciparum* using two SYBR Green-based fluorescent assays.

### Optimization of the SYBR green micro titre plate assay

**Figure 1:**
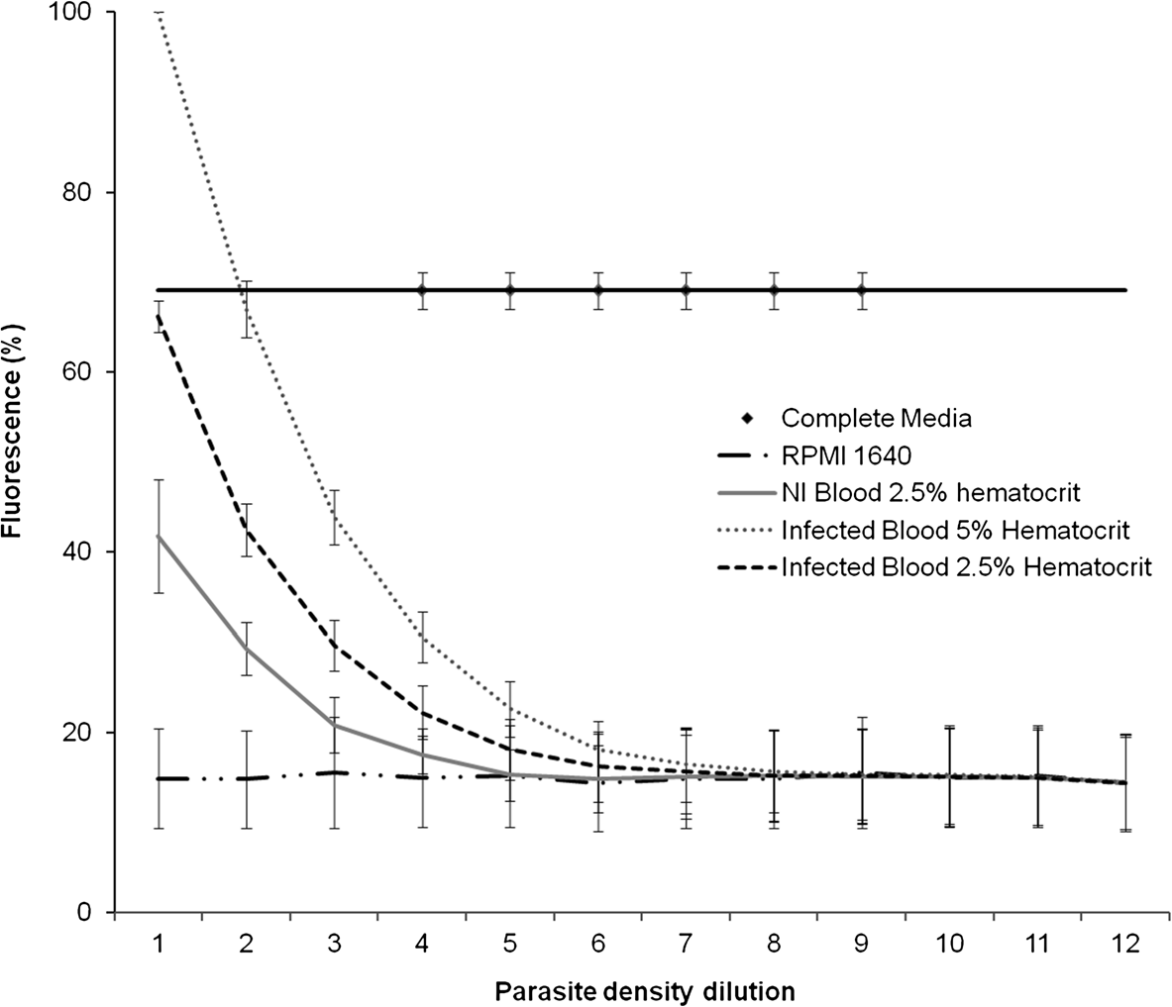
**SYBR Green fluorescence intensity as a measure of parasite density.** The SYBR Green fluorescence of parasitized blood at 5% or 2.5% haematocrit was measured at serial 2- fold dilutions in a 96 well micro titre plate assay. The correlation between parasite density and fluorescence intensity was determined. Controls included non-infected blood at 5% haematocrit, RPMI 1640 medium without Albumax and complete RMPI medium with Albumax. Error bars represent the standard error of two replicate experiments.

The SYBR green method used here is a modification of methods published previously [22,23]. Due to the non-specific nature of the double-stranded DNA intercalation by the SYBR Green dye, stringent blood washing steps were introduced to ensure complete removal of the buffy coat containing nucleated white blood cells. The SYBR Green micro titre plate-based assay was initially optimized using 2-fold serial dilutions of K1 parasite cultures at a haematocrit of 2.5 and 5% according to methods described above. Fluorescence intensities were measured on a GENios plate reader (Tecan) with excitation and emission wavelengths set at 485 nm and 535 nm respectively. Preliminary results with parasite cultures showed very poor reproducibility and little correlation between parasite density and fluorescence. Further method optimization identified the ‘complete’ RPMI medium from parasite cultures as being responsible for the variance in the results observed. The high background fluorescence was identified to the presence of Albumax® supplement in the complete media. RPMI media without Albumax showed minimal background fluorescence, and the introduction of a wash step with RPMI medium to remove the Albumax restored assay reliability and reproducibility (Figure 1).

### Optimization of the SYBR green-based flow cytometry assay

**Figure 2:**
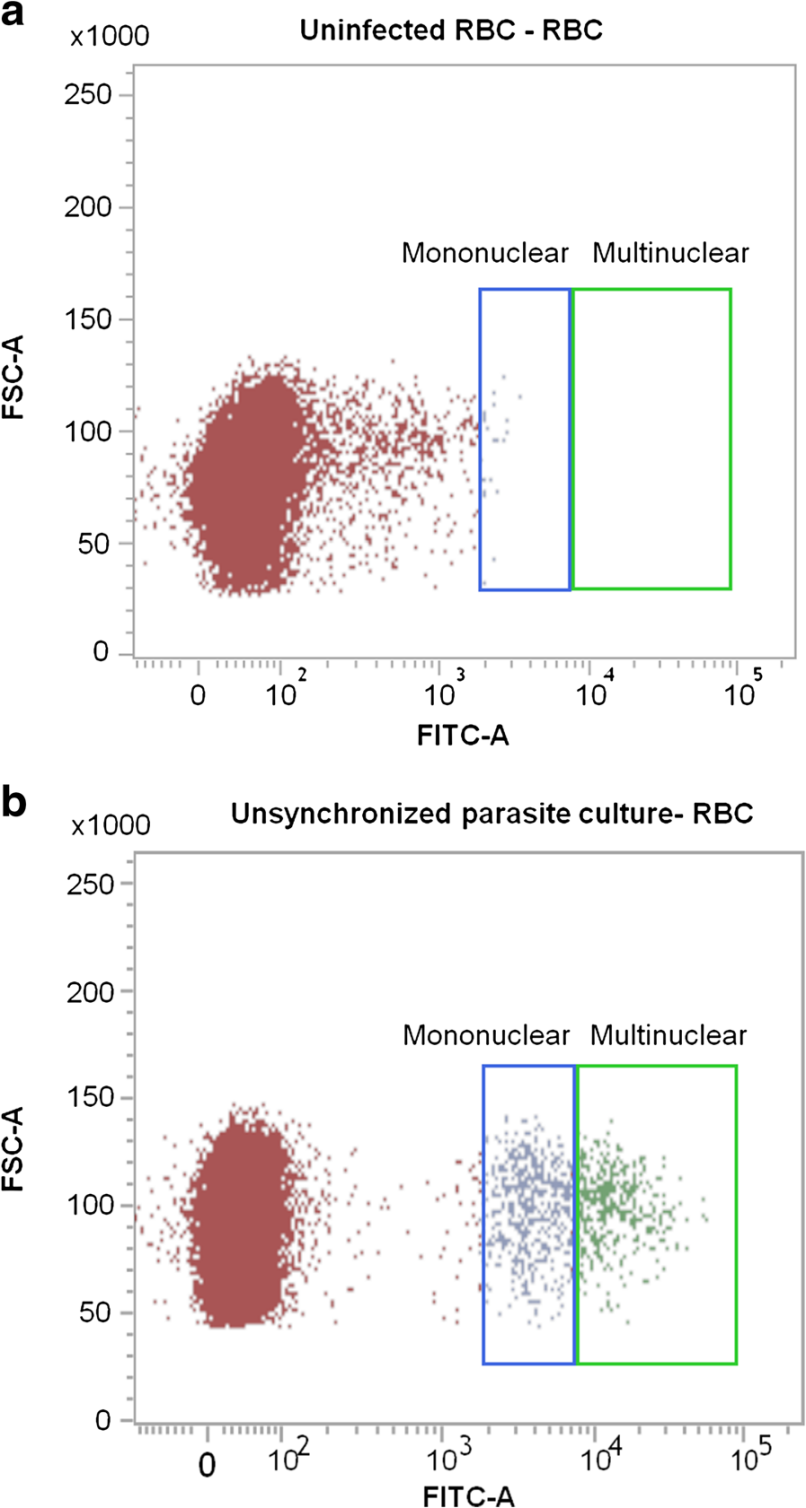
**Comparison of parasitized and uninfected blood using the SYBR Green flow cytometric assay.** Scatterplots from synchronised **(a)** uninfected and **(b)** infected cultures showing the gating strategy that enables the differentiation of mononuclear (ring/trophozoite) and multinuclear (schizont) parasite stages.

**Figure 3:**
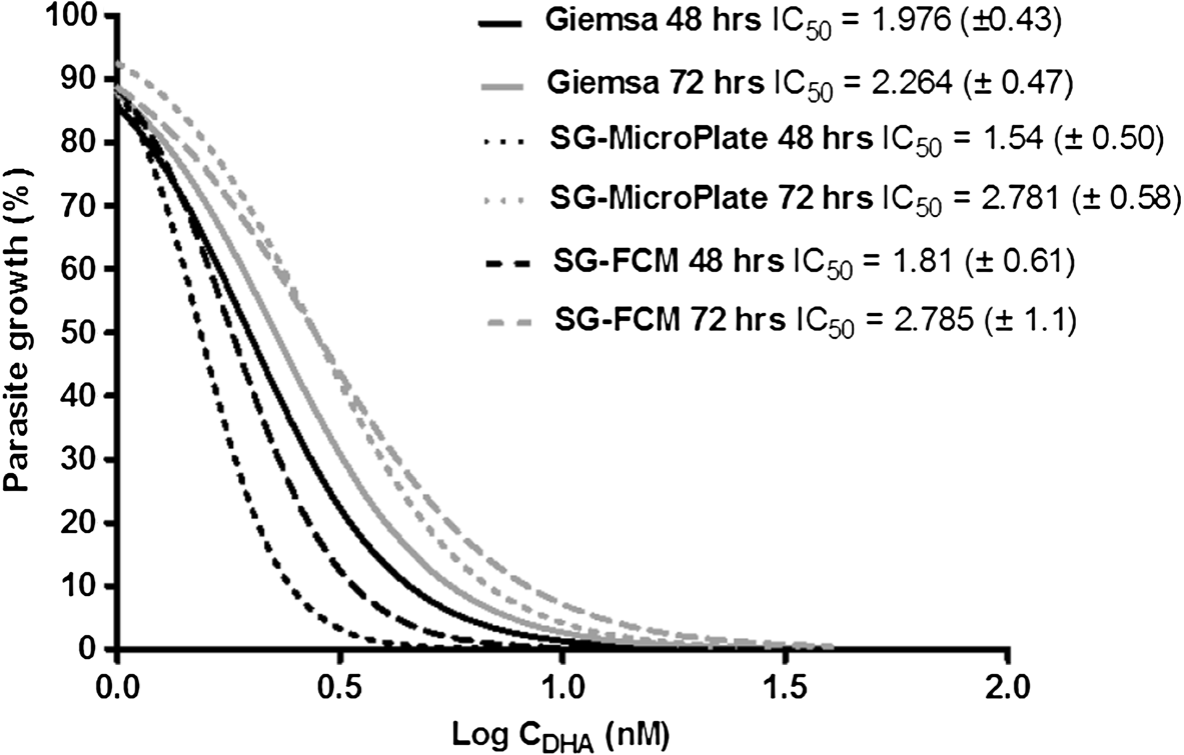
**Dose response curves for dihydroartemisinin against the K1 strain.** A set of three parallel assays: namely, Giemsa microscopic test (Giemsa), SYBR Green microtitre plate assay (SG-Microplate) and the SYBR Green flow cytometer assay (SG-FCM). Cultures were initiated at ring stage and exposed to drugs for either 48 or 72 hrs. Each curve represents data from three independent experiments.

For the flow cytometric analysis, the gating strategy was adapted as previously described [24] and permitted the differentiation between mononuclear (ring and trophozoite) and multinuclear (schizont) parasite stages in unsynchronized K1 cultures (Figure 2). Accurate determination of percentage parasitaemia was achieved using the BDFACS Verse software programme. The dose response effect of dihydroartemisinin on synchronized K1, *P. falciparum* cultures initiated at ring stage, was compared between SYBR Green flow cytometric, micro titre plate and traditional Giemsa microscopic assays (Figure 3). IC_50_ values for cultures sampled at 48 and 72 h post drug exposure were determined and compared using a one-way ANOVA (Graphpad prism). There were no significant differences between the three assays and although the IC_50_ values appear to be consistently higher at 72 hours than at the 48 hour time point for all three assays (Figure 3) this difference was not found to be significant.

### Preliminary screening of selected compounds from the LOPAC library

**Figure 4:**
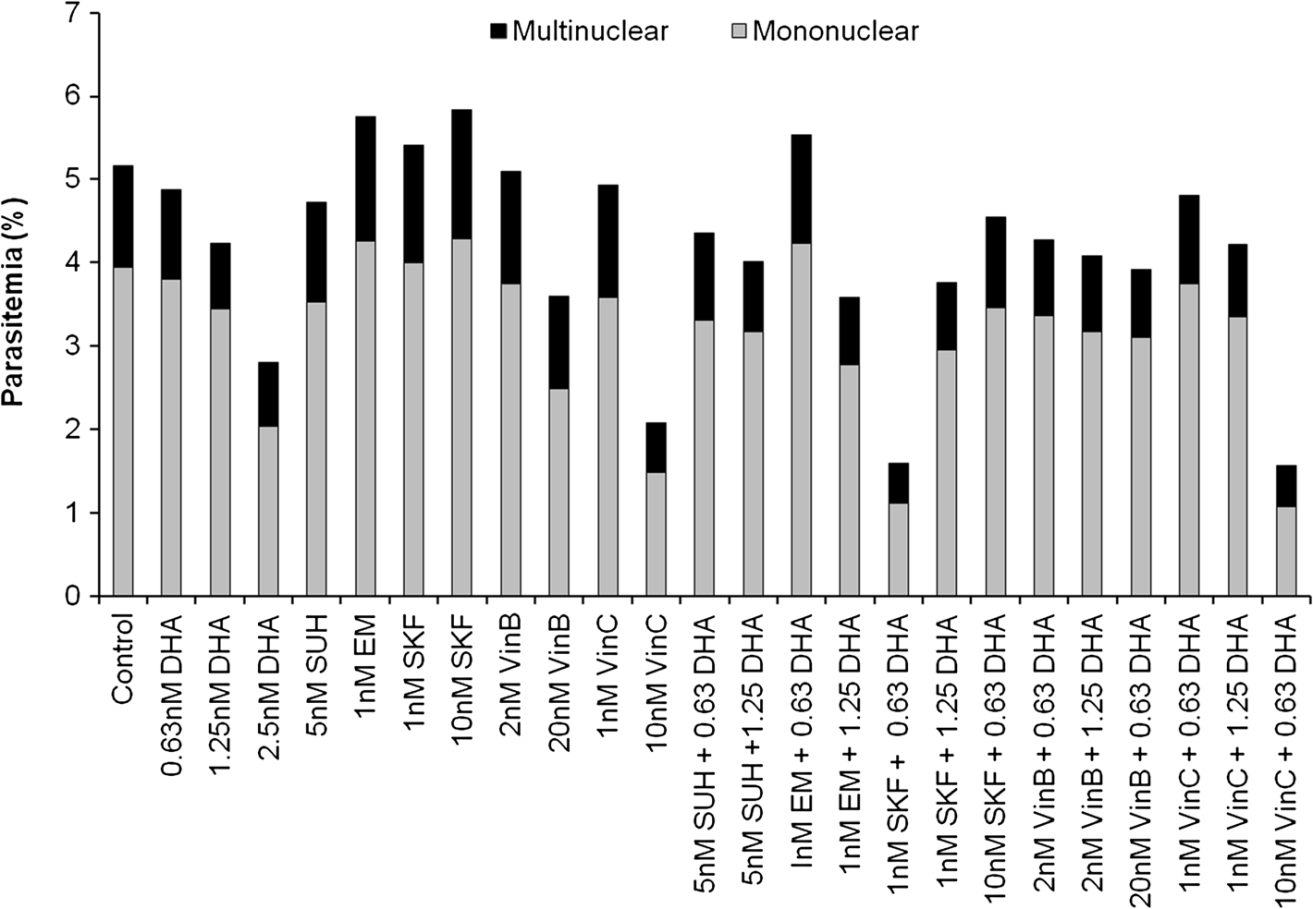
**Preliminary screen of five LOPAC compounds with dihydroartemisinin.** The LOPAC compounds Emetine dihydrochloride hydrate (EM), SKF 95282 dimaleate (SKF), S (−)-UH-301 hydrochloride (SUH), Vinblastine (VinB) and Vincristine (VinC) were either added alone or in combination with DHA for a 48 hr treatment period. All samples were analysed using the SYBR Green flow cytometric method. Parasitized blood and parasites treated with a range of DHA concentrations served as controls. Non-infected blood was also included and background fluorescence was deducted from all samples accordingly.

The LOPAC compound previously reported to have the highest anti-malarial potency (≤ 5 nM against *P. falciparum* strain 3D7) were selected for further interrogation against the K1 strain. An exemplar panel is shown in Figure 4. The nanomolar concentrations selected were based on preliminary IC_50_ values reported by Lucumi *et al*. [15], and 10-fold concentrations to accommodate variations due to the use of a drug resistant parasite strain. Dihydroartemisinin (DHA) controls, at 0.63, 1.25 and 2.5 nM, were included in the panel for comparison. A combination experiment was also carried out on the initial screen with DHA doses of 0.63 nM and 1.25 nM. Dose response experiments on the three drugs showed emetine to have the lowest IC_50_ value providing a rationale for further investigation.

### Determination of dose response curves for emetine and dihydroartemisinin

**Figure 5:**
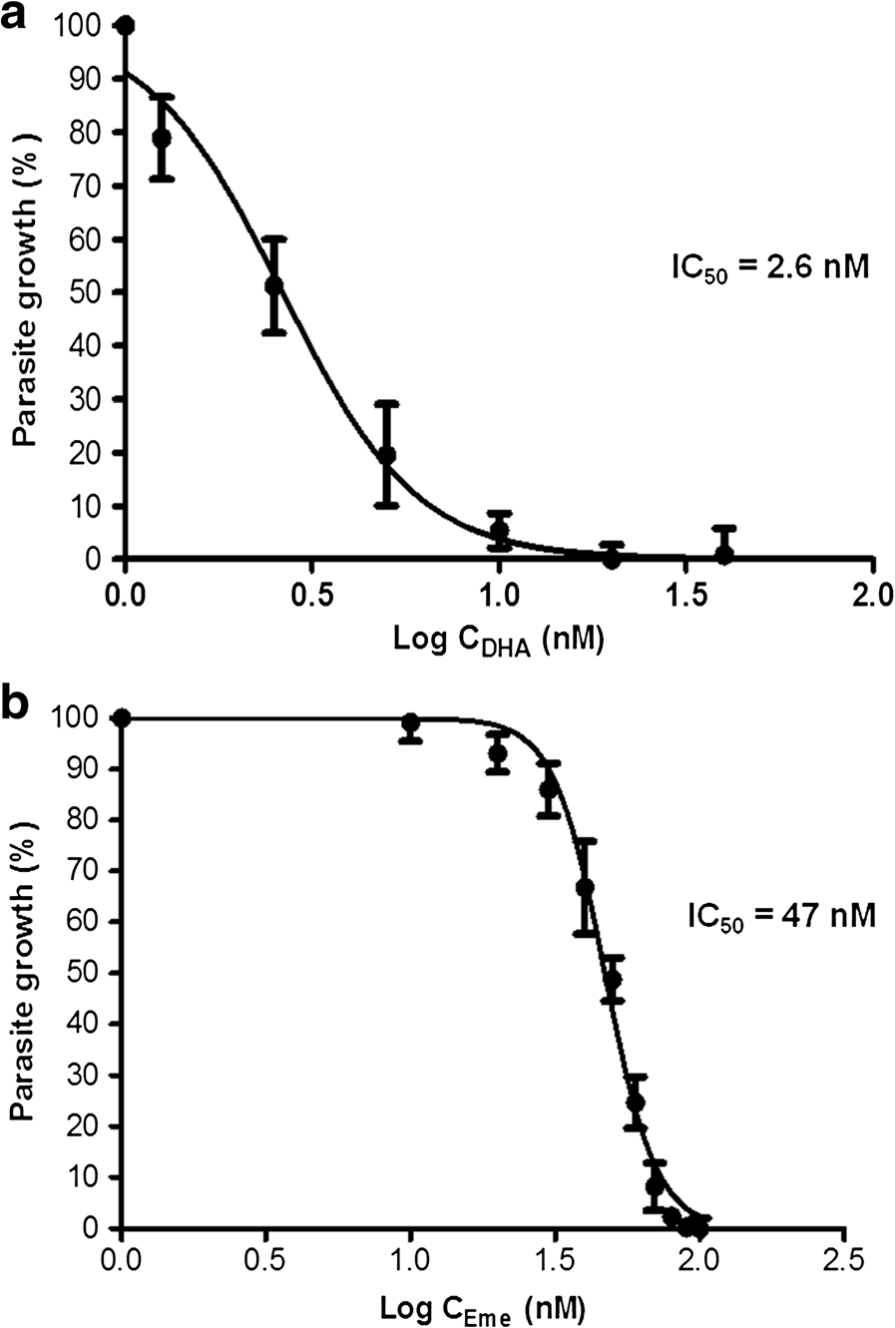
**Representative dose response curves for dihydroartemisinin (a) and emetine (b).** Parasites were treated at trophozoite stage and analysed at 48 hours using the SYBR Green flow cytometer method. IC_50_ values calculated from at least three independent experiments are also displayed.

Emetine dihydrochloride hydrate was reported to have an IC_50_ value of 1 nM on the drug sensitive 3D7 *P. falciparum* parasite strains. Preliminary screening panels from our experiments showed effective parasite suppression for the drug, both alone and in combination with DHA. Dose response curves were determined for both drugs using K1 resistant isolates and IC_50_ values of 47 ± 2.1 nM and 2.6 ± 0.41 nM established for emetine dihydrochloride hydrate and DHA, respectively (Figure 5).

### Isobologram preparation and data analysis

Drug interaction studies for emetine dihydrochloride hydrate and DHA were performed using a modification of the fixed ratios method, employing 4 fixed ratios of 4:1, 3:2, 2:3 and 1:4. IC_50_ values were determined for each compound at each ratio as described above and used to calculate fractional inhibitory concentration for each ratio using the equation below. Drug interactions were analysed both at IC_50_ and IC_90_ values.

$$\text{FIC}=\frac{\text{Fraction}\;\text{of}\;\text{drug}\;\text{concentration}\;\text{required}\;\text{to}\;\text{produce}\;\text{IC}50\;\text{when}\;\text{used}\;\text{in}\;\text{combination}}{\text{Fraction}\;\text{of}\;\text{drug}\;\text{concentration}\;\text{required}\;\text{to}\;\text{produce}\;\text{IC}50\;\text{when}\;\text{used}\;\text{alone}}$$

**Figure 6:**
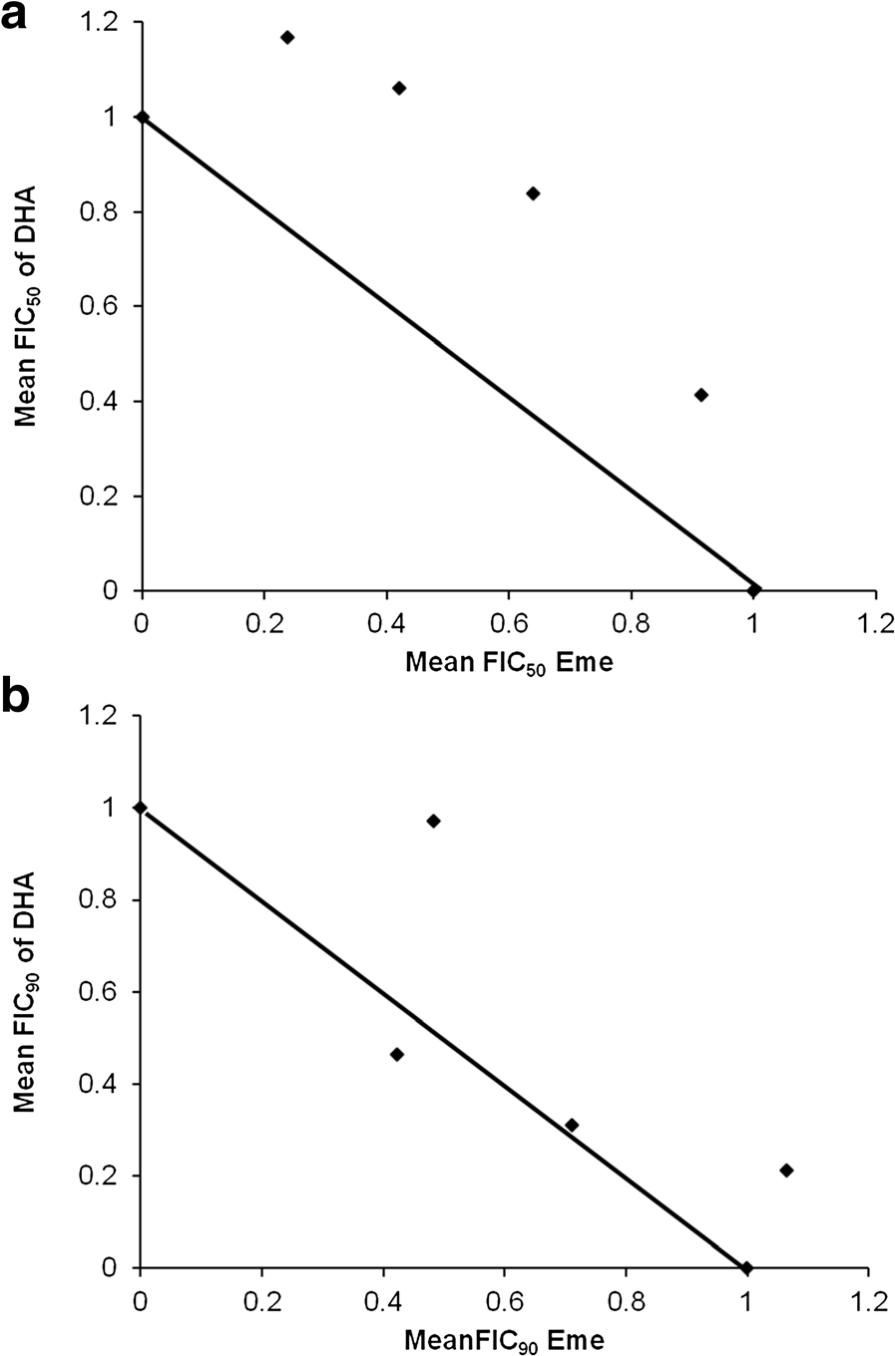
**Isobolograms showing the interaction between dihydroartemisinin and emetine.** The fixed ratio method was used to analyse the interaction between dihydroartemisinin and emetine dihydrochloride hydrate against trophozoite stage parasites. Treatments were set up in duplicate for 48 hours and analysed using the SYBR Green flow cytometer method. The FIC_50_**(a)** and FIC_90_**(b)** concentrations were determined and used to plot the isobologram.

The FIC values for DHA at each ratio were then plotted against those calculated for emetine and the isobologram trends obtained are shown in Figure 6. The convex shape of the IC_50_ isobologram (Figure 6a) indicates an antagonistic interaction while the IC_90_ plot would suggest an additive interaction (Figure 6b).

To investigate the interaction between DHA and emetine at each ratio the calculated FIC values for each compound were then added together to obtain the sum of the fractional inhibitory concentration (∑FIC) as per equation below.

$$\text{SumFIC}\left(\sum \text{FIC}\right)=\frac{\text{IC}50\;\text{of}\;A\;\text{in}\;\text{mixture}}{\text{IC}50\;\text{of}\;A\;\text{alone}}+\frac{\text{IC}50\;\text{of}\;B\;\text{in}\;\text{mixture}}{\text{IC}50\;\text{of}\;B\;\text{alone}}$$

The results from the fixed ratio experiment are presented in Table 1.

**Table 1:** **The interaction between dihydroartemisinin and emetine against *P. falciparum *(strain K1) at 6 fixed drug ratios**

**Ratio**	**Compound A (DHA) FIC**_**50**_	**Compound A (DHA) FIC**_**90**_	**Compound B (Eme) FIC**_**50**_	**Compound B (Eme) FIC**_**90**_	**∑FICs50, interaction**	**∑FICs90, interaction**
**5:0**	1	1	0	0	n/a	n/a
**4:1**	0.914829	0.97142	0.412807	0.482675	1.327636,	1.454094
**3:2**	0.638616	0.464741	0.837115	0.422492	1.475731	0.887233
**2:3**	0.421106	0.310655	1.059061	0.71155	1.480167	1.022206
**1:4**	0.238412	0.212022	1.167547	1.064742	1.405959	1.276764
**5.0**	0	0	1	1	n/a	n/a

Individual FIC_50_ and FIC_90_ values for each ratio are displayed alongside respective ∑FIC values.

All values represent an average of duplicate experiments.

### *In vitro* stage-specific effects of DHA and emetine dihydrochloride hydrate

**Figure 7:**
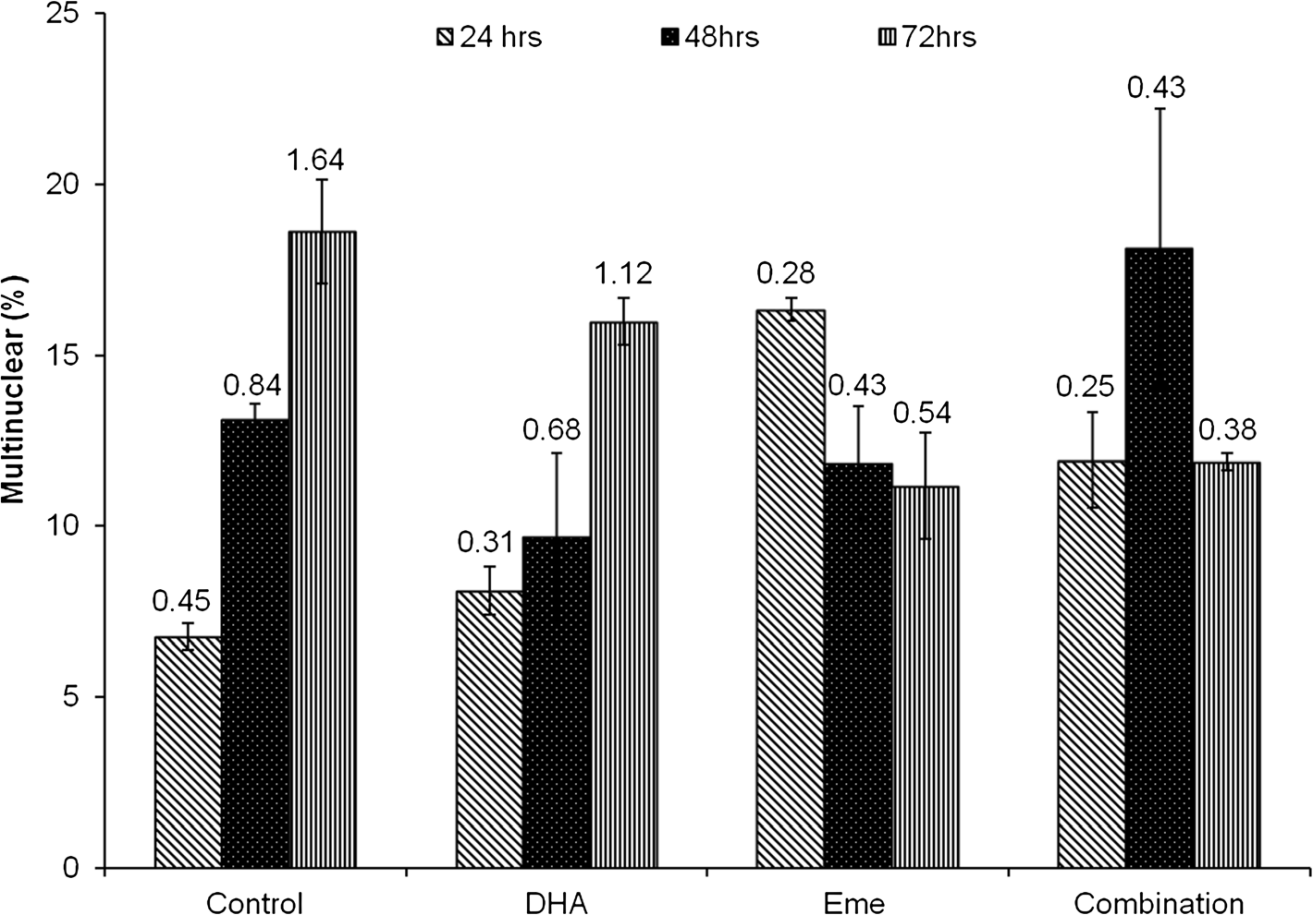
***In vitro *stage specific effects of dihydroartemisinin and emetine against *P. falciparum. *** Trophozoite stage parasites were treated with IC_50_ DHA and IC_50_ emetine either alone or in combination for a 24, 48 and 72 hour time course. The percentage of multinuclear cells as a proportion of the total parasitaemia was recorded for each condition at each time point. The total parasitaemia is indicated by the numbers above bars.

The results of the stage-specific analysis of DHA and emetine revealed variations in parasite progression pattern during the course of drug treatment. For DHA treated samples, progress into the multinucleated schizont form imitate that of the untreated control samples. However, emetine treated samples show a reversal of the pattern, representing a delay in parasite development. The combined effect of the drugs appears to display a true amalgamation of the two (Figure 7). It is also noteworthy that the overall parasitaemia of the combination is consistently lower throughout the course of drug than either of the individual component drugs.

## Discussion

The objective and rigorous investigation of the large complement of anti-malarial drug candidates that have come through preliminary screening initiatives is a high priority. The second phase of inquiry must necessarily be more refined and objective, ideally providing a reliable quantitative indication of the early sub-cellular perturbations resulting from drug activity.

The use of SYBR Green to monitor drug susceptibility exploits the fact that the parasites reside in red cells, which are devoid of a nucleus. A potential source of error would be the indiscriminate binding of SYBR Green to non-parasite DNA from white blood cells in the blood. However, the blood washing steps outlined in the methods section have helped to overcome this problem. Two SYBR Green fluorescence-based assays enabling the accurate and reproducible estimation of the effects of the drugs on the intracellular parasite stages were optimized. The higher throughput 96 well microtitre plate fluorescent assay tends to overestimate parasitaemia due to fluorescence from extra-erythrocytic parasite DNA. Optimizations achieved increased method reproducibility by identifying the very significant contribution of the albumax supplement from the parasite culture medium to variations in background fluorescence. The SYBR Green flow cytometry method uses a single tube format for analysis and offers a robust, albeit lower throughput route for the more in depth second phase inquiry of anti-malarial drug candidates selected from preliminary screens. Furthermore, stage-specific perturbations introduced by the drug could be accurately monitored. Hence in combination, the two methods described here would be valuable tools to screen and investigate the anti-malarial efficacy of compound libraries.

Following on from the work of Lucumi *et al.*[15], five compounds were selected from preliminary screens carried out in, namely emetine dihydrochloride hydrate, SKF 95282 dimaleate, S (−)-UH-301 hydrochloride, Vinblastine® and Vincristine®. A multi-drug resistant *P. falciparum* parasite line (K1) was used to derive preliminary dose response curves. Emetine dihydrochloride hydrate was taken forward for second phase investigation based on the dose response curves obtained. The drug exhibited potent anti-malarial properties at nanomolar concentrations (~47 nM) on the multi-drug resistant parasite line K1 (1 nM in drug sensitive parasite lines [9]), justifying further investigation into its role as a stand-alone anti-malarial drug.

Emetine dihydrochloride is an anti-protozoal drug derived from the root of *Carapichea ipecacuanha* (Ipecac®). The drug was previously used in the treatment of invasive intestinal amoebiasis and amoebic liver abscess [25]. Its use as a potent intestinal and tissue amoebicide was restricted due to side effects of nausea and vomiting induced by irritant effects on the intestinal mucosa, following oral administration. Furthermore, the higher doses required for tissue amoebicidal activity showed cardiotoxic effects in a proportion of the patients treated [25]. The concentration dependent negative inotropic and chronotropic effects are thought to be mediated via the blocking of L-type calcium channels in the heart [26]. Although ECG changes including T-wave inversion and prolongation of the Q-T interval are reported at higher dose ranges (total dose > 1gm), cardiovascular function usually returns to normal [27]. Unfortunately, while the cardiotoxicity studies on emetine define the side effects in relation to the administered dose, very little is known regarding the plasma concentrations after therapeutic administration or indeed the proportion of drug bound to plasma proteins. Studies in rats and dogs have shown tissue concentrations in the liver and kidney to be higher than in plasma. However, significant species and strain-specific variability in the susceptibility of experimental animals to emetine toxicity preclude the extrapolation of these findings to humans [27].

A synthetic modification of the drug, dehydroemetine (Roche), which structurally differs from the dihydroemetine hydrate only in a double bond next to the ethyl substituent, is reported to retain its anti-amoebicidal properties while producing fewer side effects [28]. This development achieved a major breakthrough in the treatment of amoebiasis with seven-day treatment regimes (1 mg/kg body weight) resulting in fewer side effects due to reduced accumulation in tissues [28,29]. Early radio tracer studies by Schwartz in 1965 comparing excretion of emetine dihydrochloride and dehydroemetine reported 67% and 91% clearance respectively, 3 days after treatment, possibly explaining the reduced side effects of the latter [30]. The replacement of emetine with the much safer metronidazole meant that further research into the drug was not actively pursued. More recent work comparing *in vitro* data on emetine for Entamoeba species report IC_50_ values ranging from 26 to 60 μM [30]. In contrast, the *in vitro* IC_50_ data reported for malaria ranges from 5–50 nM, making an objective argument for the further investigation of emetine as a repositioned drug in malaria.

To permit further dose reductions in the drug, we further investigated the role of emetine dihydrochloride hydrate as a candidate for artemisinin combination therapy. Data from preliminary drug interaction studies of emetine dihydrochloride hydrate and DHA show the effect to range from additive to mildly antagonistic depending on the dose ratios used. While the FIC_50_ isobolograms show an antagonistic trend, the FIC_90_ isobolograms follow an additive trend. The sum 50% and 90% fractional inhibitory concentration (∑FIC _50. 90_) of the interaction of emetine dihydrochloride hydrate and DHA against the K1 strain of *P. falciparum* ranged from 0.88-1.48. ∑FIC values predict the drug ratios to fall mainly within the additive range when applying criteria used by Bhattacharya *et al.* (i.e. ∑FIC < 1 synergism, ∑FIC ≥ 1 and < 2 additive, interaction, ∑FIC ≥ 2 and < 4 slight antagonism, ∑FIC ≥ 4 marked antagonism). When the more stringent criteria used by Abiodun *et al*. [31], are applied (i.e. ∑FIC < 0.8 = synergism, ∑FIC ≥ 0.8-1.4 = additive, ∑FIC ≥ 1.4 = antagonistic) the drug ratios fall within the additive to mildly antagonistic range. Vivas *et al.*[32] interpret interactivity as ∑FIC cut off values for synergy or antagonism being < or > 1 respectively, with ‘additivity’ defined as ∑FICs = 1. However, the very narrow definition of ‘additivity’ here could result in the exclusion of potentially effective compounds combination with very few hits identified in this category.

Assessing *in vitro* interactions between anti-malarials has gained increased significance with increasing evidence of combination therapies postponing resistance [18]. The widely used checkerboard and fixed-drug ratio methods are reliant on the predetermination of IC_50_ values of the component drugs [32-34]. The latter has advantages over the former because the dose response curves depend on drug concentration ratios calculated on the basis of 100-0% parasite inhibition which permits a more rigorous and accurate calculation of regression curve fit and IC_50_ values. Variations in FIC cut off values proposed in published literature however, provide very little guidance on standardized interpretations for these parameters. A review of the current literature on interpretation of drug interaction data highlights many pitfalls and inaccuracies [35,36]. For diseases like malaria, combinatorial drug regimes will ensure delaying the onset of resistance and the search for suitable partner drugs will be a high priority. Hence, the surprising lack of consensus and standardized methods for interpreting drug interaction data is rather disconcerting. Furthermore, simplistic mechanistic deductions based on parasite clearance may result in overlooking important candidates merely because they are judged mildly antagonistic using current methods which define synergy based on combined anti-parasitic potency being higher than the individual potencies added together. Data from preliminary stage-specific experiments where the drug was added to synchronized cultures in the trophozoite phase, show distinct differences in the progression of the life cycle through 24, 48 and 72 h (Figure 7). The inevitable advantages afforded by a two pronged attack in delaying the onset of resistance and perhaps reducing therapeutic doses of individual drugs and hence their side effects, may need to be considered in an objective manner prior to simplistically labelling candidates as antagonistic, additive or synergistic.

The most successful anti-malarials to date have indeed been plant derived molecules (quinolones/artemisinins). In emetine, yet another opportunity is presented to investigate a natural product which has been already been shown to be amenable to chemical modification and toxicity reduction. Malaria is a deadly disease with a complex clinical presentation. The hepatic concentration of emetine could be potentially exploited to address the lack of treatment options to destroy the dormant liver stages of the parasite. Its nanomolar anti-malarial efficacy makes it a useful reserve treatment option for cerebral malaria where in-hospital patient monitoring is routine. Since most of the pharmacokinetic published work on emetine was carried out in the 1960’s, reliable quantitative data on serum/tissue levels are lacking. Revisiting this area of research with the benefit of current post-genomic technologies would enable the quantitative definition of these parameters and perhaps permit the safe use of the drug within narrow therapeutic windows.

## Abbreviations

ACT: Artemisinin combination therapy; ANOVA: Analysis of variance; DHA: Dihydroartemisinin; DMSO: Dimethyl sulfoxide; IC50: Inhibitory concentration 50; Eme: Emetine; FIC: Fractional inhibitory concentration; LOPAC: Library of Pharmaceutically Active Compounds; FITC: Fluorescein isothiocyanate; NINDS: National Institute of Neurological Disorders and Stroke; PBS: Phosphate buffered saline; SG-FCM: SYBR Green flow cytometry; WHO: World Health Organization.

## Competing interests

The authors declare they have no competing interests.

## Authors’ contributions

NN, HM, FK, MR conceived and designed the experiment. HM, MI carried out the laboratory work. HM analysed the data. NN, FK contributed to reagents. NN, HM wrote the paper. All authors had full access to all the data in the study and read and approved the manuscript.

## References

[B1] Kremsner PG Krishna S Antimalarial combinations Lancet 2004 364 285 294 10.1016/S0140-6736(04)16680-4 15262108

[B2] World Health Organization Global report on antimalarial drug efficacy and drug resistance: 2000–2010 2010 Geneva: WHO

[B3] Dondorp AM Yeung S White L Nguon C Day NPJ Socheat D Von Seidlein L Artemisinin resistance: current status and scenarios for containment Nat Rev Micro 2010 8 272 280 10.1038/nrmicro2331 20208550

[B4] Jambou R Le Bras J Randrianarivelojosia M Pitfalls in new artemisinin-containing antimalarial drug development Trends Parasitol 2011 27 82 90 10.1016/j.pt.2010.09.004 21030307

[B5] Walsh JJ Coughlan D Heneghan N Gaynor C Bell A A novel artemisinin-quinine hybrid with potent antimalarial activity Bioorg Med Chem Lett 2007 17 3599 3602 10.1016/j.bmcl.2007.04.054 17482816

[B6] White NJ Antimalarial drug resistance J Clin Invest 2004 113 1084 1092 15085184 10.1172/JCI21682 PMC385418

[B7] Ashburn TT Thor KB Drug repositioning: identifying and developing new uses for existing drugs Nat Rev Drug Discov 2004 3 673 683 10.1038/nrd1468 15286734

[B8] Ekins S Williams AJ Krasowski MD Freundlich JS In silico repositioning of approved drugs for rare and neglected diseases Drug Discov Today 2011 16 298 310 10.1016/j.drudis.2011.02.016 21376136

[B9] Walker SL Waters MF Lockwood DN The role of thalidomide in the management of erythema nodosum leprosum Lepr Rev 2007 78 197 215 18035771

[B10] Fink HA Mac Donald R Rutks IR Nelson DB Wilt TJ Sildenafil for male erectile dysfunction: a systematic review and meta-analysis Arch Intern Med 2002 162 1349 1360 10.1001/archinte.162.12.1349 12076233

[B11] DiMasa JA Hansen RW Grabowski HG Lasagna L Cost of innovation in the pharmaceutical industry J Health Econ 1991 10 107 142 10.1016/0167-6296(91)90001-4 10113009

[B12] Tartaglia LA Complementary new approaches enable repositioning of failed drug candidates Expert Opin on Investig Drugs 2006 15 1295 1298 10.1517/13543784.15.11.1295 17040191

[B13] Nzila A Ma Z Chibale K Drug repositioning in the treatment of malaria and TB Future Med Chem 2011 3 1413 1426 10.4155/fmc.11.95 21879845

[B14] Burrows JN Leroy D Lotharius J Waterson D Challenges in antimalarial drug discovery Future Med Chem 2011 3 1401 1412 10.4155/fmc.11.91 21879844

[B15] Lucumi E Darling C Jo H Napper AD Chandramohandas R Fisher N Shone AE Jing H Ward SA Biagini GA DeGrado WF Diamond SL Greenbaum DC Discovery of potent small molecule inhibitors of multi drug-resistant *Plasmodium falciparum* using a novel miniaturised high throughput luciferase-based assay Antimicrob Agents Chemother 2010 54 3579 3604 10.1128/AAC.00431-10 PMC2934977 20547797

[B16] Adjuik M Agnamey P Babiker A Borrmann S Brasseur P Cisse M Cobelens F Diallo S Faucher JF Garner P Gikunda S Kremsner PG Krishna S Lell B Loolpapit M Matsiegui PB Missinou MA Mwanza J Ntoumi F Olliaro P Osimbo P Rezbach P Some E Taylor WR Amodiaquine-artesunate versus amodiaquine for uncomplicated *Plasmodium falciparum* malaria in African children: a randomised multicentre trial Lancet 2002 359 1365 1372 10.1016/S0140-6736(02)08348-4 11978332

[B17] Mutabingwa TK Anthony D Heller A Hallett R Ahmed J Drakeley C Greenwood BM Whitty CJ Amodiaquine alone, amodiaquine + sulfodoxine-pyremethamin, amodiaquine + artesunate and artemether-lumefantrine for outpatient treatment of malaria in Tanzanian children: a four armed randomised effectiveness trial Lancet 2005 365 1474 1480 10.1016/S0140-6736(05)66417-3 15850631

[B18] World Health Organization Antimalarial drug combination therapy (WHO/CDS/RBM/2001.35) 2001 Geneva: WHO

[B19] Read M Hyde JE Simple in vitro culture of the malaria parasite *Plasmodium falciparum* (erythrocytic stages): suitable for large-scale preparations Protocols in Molecular Parasitology. (Hyde JE Ed.). Volume 21 1993 43 55 10.1385/0-89603-239-6:43 8220733

[B20] Trager W Jensen JB Human malaria parasites in continuous culture Science 1976 193 673 675 10.1126/science.781840 781840

[B21] Bhattacharya A Mishra LC Sharma M Awasthi SK Bhasin VK Antimalarial pharmacodynamics of chalcone derivatives in combination with artemisninin against *Plasmodium falciparum in vitro* Eur J Med Chem 2009 44 3388 3393 10.1016/j.ejmech.2009.02.008 19269069

[B22] Vossen MG Pferschy S Chiba P Noedl H The SYBR Green I malaria drug sensitivity assay: performance in low parasitaemia samples Am J Trop Med Hyg 2010 82 398 401 10.4269/ajtmh.2010.09-0417 20207863 PMC2829899

[B23] Rason MA Randriantsoa T Andrianantenaina H Ratsimbasoa A Menard D Performance and reliability of the SYBR Green 1 based assay for the routine monitoring of susceptibility of *Plasmodium falciparum* in clinical isolates Trans R Soc Trop Med Hyg 2008 102 346 351 10.1016/j.trstmh.2008.01.021 18321546

[B24] Karl S Wong RPM St Pierre TG Davis TME A comparative study of a flow cytometry based assessment of *in vitro Plasmodium falciparum* drug sensitivity Malar J 2009 8 294 10.1186/1475-2875-8-294 20003396 PMC2799432

[B25] Lasserre R Treatment of amoebiasis Phil J Microbiol Infect Dis 1979 8 1 1

[B26] Lemmens-Gruber R Karkhaneh A Studenik C Heistracher P Cardiotoxicity of emetine dihydrochloride by calcium channel blockade in isolated preparations and ventricular myocytes of guinea-pig hearts Brit J Pharmacol 1996 117 377 383 10.1111/j.1476-5381.1996.tb15202.x 8789394 PMC1909259

[B27] Yang WC Dubick MW Mechanism of Emetine toxicity Pharmacol Ther 1980 10 15 26 10.1016/0163-7258(80)90007-8 6996003

[B28] Dempsey JJ Salem HH An enzymatic electrocardiographic study on toxicity of dehydroemetine Brit Heart J 1966 28 505 511 10.1136/hrt.28.4.505 5942470 PMC459078

[B29] Chintana T Sucharit P Mahakittikun V Siripanth C Suphadtanaphongs W In vitro studies on the sensitivity of local *Entamoeba histolytica* to anti-amoebic drugs Southeast Asian J Trop Med Public Health 1986 17 591 594 2883732

[B30] Bansal D Sehgal R Chawla Y Mahajan RC Malla N In vitro activity of antiamoebic drugs against clinical isolates of Entamoeba histolytica and Entamoeba dispar Ann Clin Microbiol Antimicrob 2004 3 27 31 10.1186/1476-0711-3-27 15610563 PMC544836

[B31] Abiodun OO Brun R Sergio W *In vitro* interaction of artemisinin derivatives or the fully synthetic peroxidic antimalarial OZ277 with thapsigargin in *P. falciparum* strains Malar J 2013 12 43 10.1186/1475-2875-12-43 23368889 PMC3566918

[B32] Vivas L Rattray L Stewart LB Robinson BL Fugmann B Haynes RK Peters W Croft SL Antimalarial efficacy and drug interactions of the novel semi-synthetic endoperoxide artemisone *in vitro* and *in vivo* J Antimicrob Chemother 2007 59 658 665 10.1093/jac/dkl563 17337512

[B33] Canfield CJ Pudney M Gutteridge WE Interactions of atovaquone with other antimalarial drugs against *Plasmodium falciparum in vitro* Exp Parasitol 1995 80 373 381 10.1006/expr.1995.1049 7729473

[B34] Fivelman QL Adagu IS Warhurst DC Modified fixed-ratio isbologram method for studying *in vitro* interactions between atovoquone and proguanil or dihydroartemisinin against drug-resistant strains of *Plasmodium falciparum* Antimicrob Agents Chemother 2004 48 4097 4102 10.1128/AAC.48.11.4097-4102.2004 15504827 PMC525430

[B35] Bennerstrom JL Ellis WY Milhouse WK Eaton RM Chou TC, Rideout DC Antimalarial synergism and antagonism Synergism and Antagonism in Chemotherapy 2001 New York: Academic Press, Inc 183 222

[B36] Bell A Antimalarial synergism and antagonism: mechanistic and clinical significance FEMS Microbiol Lett 2005 253 171 184 10.1016/j.femsle.2005.09.035 16243458

